# Measuring appropriateness of diagnostic imaging: a scoping review

**DOI:** 10.1186/s13244-023-01409-6

**Published:** 2023-04-13

**Authors:** Felix Walther, Maria Eberlein-Gonska, Ralf-Thorsten Hoffmann, Jochen Schmitt, Sophia F. U. Blum

**Affiliations:** 1 Center for Evidence-Based Healthcare, University Hospital Carl Gustav Carus and Faculty of Medicine Carl Gustav Carus, Technische Universität Dresden, Fetscherstr. 74, 01307 Dresden, Germany; 2 Quality and Medical Risk Management, University Hospital Carl Gustav Carus, Technische Universität Dresden, Dresden, Germany; 3 Institute and Polyclinic for Diagnostic and Interventional Radiology, University Hospital Carl Gustav Carus, Technische Universität Dresden, Dresden, Germany

**Keywords:** Appropriateness, Indication, Diagnostic imaging, Key performance indicator, Audit

## Abstract

In radiology, the justification of diagnostic imaging is a key performance indicator. To date, specific recommendations on the measurement of appropriateness in diagnostic imaging are missing. To map the study literature concerning the definition, measures, methods and data used for analyses of appropriateness in research of diagnostic imaging. We conducted a scoping review in Medline, EMBASE, Scopus and the Cochrane Central Register of Controlled Trials. Two independent reviewers undertook screening and data extraction. After screening 6021 records, we included 50 studies. National guidelines (*n* = 22/50) or American College of Radiology Appropriateness Criteria (*n* = 23/50) were used to define and rate appropriateness. 22/50 studies did not provide methodological details about the appropriateness assessment. The included studies varied concerning modality, amount of reviewed examinations (88–13,941) and body regions. Computed tomography (27 studies, 27,168 examinations) was the most frequently analyzed modality, followed by magnetic resonance imaging (17 studies, 6559 examinations) and radiography (10 studies, 7095 examinations). Heterogeneous appropriateness rates throughout single studies (0–100%), modalities, and body regions (17–95%) were found. Research on pediatric and outpatient imaging was sparse. Multicentric, methodologically robust and indication-oriented studies would strengthen appropriateness research in diagnostic imaging and help to develop reliable key performance indicators.

**Supplementary Information:**

The online version contains supplementary material available at 10.1186/s13244-023-01409-6.

## Key points

- National guidelines or the American College of Radiology Appropriateness criteria were used to rate the appropriateness of diagnostic imaging.
- Evidence on the appropriateness concerning pediatric/adolescent imaging and outpatient settings is sparse.
- Heterogeneous appropriateness rates were reported throughout all body regions and modalities.
- Appropriateness across studies is not comparable because of non-standardised and monocentric data acquisition.
- Methodologically robust and indication-oriented appropriateness research is needed to improve further analyses.

**Supplementary Information:**

The online version contains supplementary material available at 10.1186/s13244-023-01409-6.

## Introduction

For medical services with particular risks of complications, the benefits of a procedure must outweigh the harmful factors. In radiology, appropriateness is the key element in the justification of diagnostic imaging. Both, the American College of Radiology (ACR) and the European Society of Radiology (ESR) define appropriateness of a radiological procedure by the evidence-based advantageousness of the risk–benefit ratio [1, 2].

An indication for a radiological procedure includes two stages. A referring physician provides the medical indication (referral) and the radiologist justifies or denies the provision of the radiological procedure. Appropriateness, as used in this review, refers to the process of justifying a radiological examination through careful consideration of the risks and benefits associated with the procedure. Improper patient selection causes under-, over- or misuse of radiological procedures [3]. Underuse is critical because of the risk of missing important diagnoses and a resulting delay in patient treatment with consecutive later or wrong diagnosis and treatment. Misuse bears the risk of excess radiation exposure and/or double investigation with consecutive resource waste. Overuse means that examinations are performed without therapeutic implications. Overuse may cause unnecessary radiation exposure and/or an overload of referrals which may lead to the delay of other urgent radiological procedures [3–5]. Resource allocation is crucial. Especially patients in countries with low density of large-scale equipment encounter longer waiting times [6].

Costs of radiological procedures are one of many variables in a healthcare system and a balanced justification is needed [2, 7, 8]. However, the ESR emphasises clearly that an individual justification must not be influenced by costs but only a favorable risk–benefit ratio [2].

Recent literature focuses on the appropriateness of referrals and certain indications like low back pain [9], which represents the quality of the medical indication given by a referrer [10].

As the ESR stated in 2020, the monitoring of undertaken diagnostic imaging, particularly high-dose studies, could serve as a key performance indicator for auditing radiation protection [11]. Their statement is rather general and specific recommendations on the realization are missing, as well in the Esperanto Guide to Clinical Audit as in other publications [12–14]. Therefore, our review focused on analysing the appropriateness of the diagnostic imaging that was performed, with radiologists serving as the gatekeepers responsible for determining whether the imaging is necessary. As part of our review we identified the following key questions:

(i) What is the definition of appropriateness in diagnostic radiology in different study settings?
(ii) What are the measures and results of appropriateness in diagnostics in different study settings?
(iii) Which methods are used to measure appropriateness in radiological diagnostics?
(iv) Which data are used to measure appropriateness in radiological diagnostics?

## Methods

We conducted a *Scoping Review* to answer the objectives mentioned above using the updated guideline for Systematic Scoping Reviews [15]. Scoping reviews are indicated if a research field has not yet been systematically reviewed and the topic is complex*.* This mapping of evidence initially helps to identify entry points and relevant issues for specific evidence syntheses (including systematic reviews) [16, 17]. The results of scoping reviews are usually analyzed by using descriptive statistical methods and can then be visualized and presented by evidence mapping without critical appraisal [15]. For reporting, we applied the PRISMA-ScR Checklist [18].

### Inclusion and exclusion criteria based on the population–concept–context (PCC) framework

Based on the pre-defined inclusion and exclusion criteria (Table 1), we published the protocol (Additional file 1: Supplementary material 1) of this scoping review online at the Center for Open Science Framework (OSF). We excluded radiotherapy, screening studies and animal studies. Due to missing details and or research design we excluded commentaries, case reports and conference papers.

**Table 1:** Inclusion and exclusion criteria based on the PCC (Population–Concept–Context) framework

Criteria	Inclusion criteria	Exclusion criteria
Population	Diagnostic patients: - Undergoing radiological diagnostics - Of any age, (co)morbidity and sex	Screening programs (e.g., breast, lung, prostate screening) Radiotherapy (e.g., radiation) Animal studies
Concept	Studies analysing appropriate and targeted use of radiological diagnostics Studies measuring appropriate indication of radiological diagnostics	Clinical practice guidelines
Context	Single studies as well as aggregated evidence (systematic reviews, meta-analyses)	
Publication type and language	Published journal articles available or articles not yet peer-reviewed Articles available as full text No language restrictions	Commentaries, case reports, conference papers

### Search strategy

A systematic literature search (19/07/2021) was conducted in *Medline*, *EMBASE* (via OVID), *Cochrane Central Register of Controlled Trials* and *Scopus*. The search strategy contained pre-defined keywords, search and MESH terms (Additional file 1: Supplementary material 2). The published protocol in OSF included five key papers [19–23] to validate the search strategy. One of these five key papers was the study of Cristofaro [19], which turned out to analyze radiology requests but not the appropriateness of the actual diagnostic imaging. As our scoping review focuses on studies with actual radiological diagnostic procedures and omits studies about the quality of referrals, we removed this study from screening, diverging from the initial protocol uploaded at OSF. In addition, already published (systematic) reviews as well as the reference lists of the included articles (backward citation tracking) and articles citing these were screened (forward citation tracking via Scopus).

### Study selection

After removing duplicates using Endnote V9, the results were screened by two independent reviewers (F.W., S.B.) at title-/abstract and full-text level using Rayyan (https://rayyan.qcri.org/). In the case of diverging ratings of relevance on the full-text level, both reviewers reached a consensus.

### Extraction and synthesis of relevant content

One reviewer performed the data extraction and was checked by the other reviewer. The extraction content included study characteristics, methods and appropriateness results. Many guidelines like the ACR appropriateness criteria (AC) differentiate between usual/full appropriateness, maybe/moderate appropriateness and not appropriate [1]. To keep a dichotomous and interpretable synthesis, and to include all results that have already been aggregated, we interpreted results of a maybe/moderate appropriateness as usual/fully appropriate in the overall rating. If the results were already aggregated to dichotomous results by the study authors, we extracted the results as reported by the literature. If information was missing or results were indeterminate, we highlighted these results as indeterminate/not applicable.

Both reviewers extracted the data in a piloted standardized data extraction spreadsheet (Excel) using five included articles to perform possible modifications before the extraction of all included studies. We synthesized the study results according to the different modalities, applied guidelines and body regions. We summarized the descriptive appropriateness results aggregated by modality and body region.

## Results

**Fig. 1:**
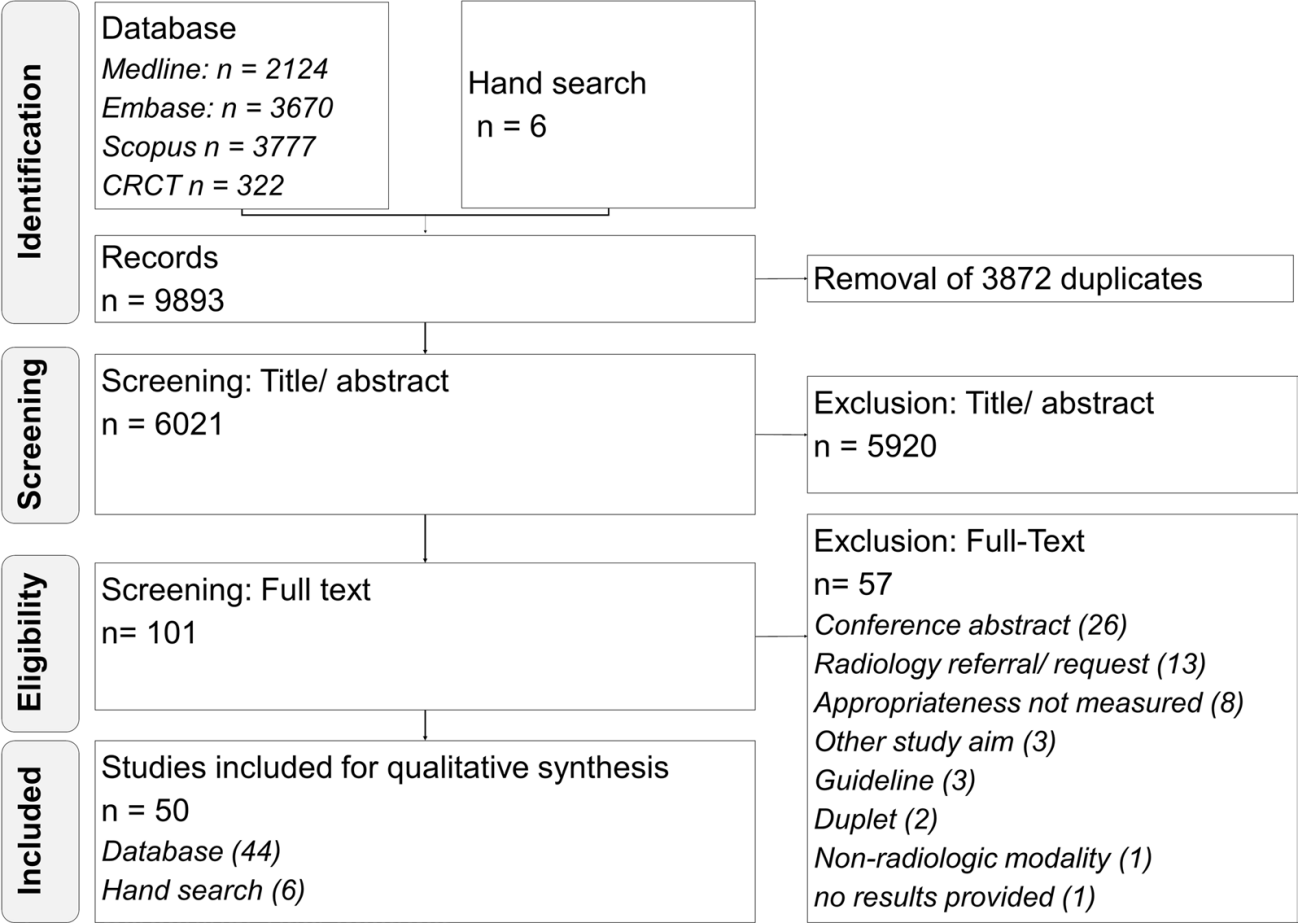
The Preferred Reporting Items for Systematic reviews and Meta-Analyses (PRISMA) extension for Scoping Reviews Flowchart. The Preferred Reporting Items for Systematic reviews and Meta-Analyses (PRISMA) extension for Scoping Reviews Flowchart shows the identified and screened records on title-abstract and full-text basis and the number of finally included studies for data extraction

After screening 6,021 records, 101 full-text publications were screened for eligibility (Fig. 1). The most frequent reasons for exclusion at full-text level (Additional file 1: Supplementary material 3) were conference abstracts (*n* = 26) [24–49] and studies exclusively investigating the appropriateness of requests for diagnostic imaging (*n* = 13) [19, 50–61]. Finally, 50 studies met the predefined inclusion criteria and were included in this scoping review [20–23, 62–107].

Table 2 describes the detailed characteristics of the included studies summarized below. Most of the studies were conducted in the USA (16/50), Canada (6/50), Australia (3/50) or Western Europe, particularly the United Kingdom (4/50), Italy (4/50), Spain (3/50), Germany (2/50) or Finland (2/50) (Table 2). The oldest study was published in 1994 [90]. The vast majority of studies (39/50) were published within the past ten years [20–23, 64–71, 74–77, 79–83, 85, 87–89, 91, 92, 94, 96, 98–107]. Most of the studies were fully (35/40) or partially (5/40) undertaken in inpatient settings and included varying numbers of participants (1st Percentile: 225, Median: 503, 3rd percentile: 1295). The most common modality was computed tomography (32/50), followed by magnetic resonance imaging (19/50), radiography (15/50) and ultrasound (3/50).

**Table 2:** Characteristics of included studies

Ref.	Year	Country	Population age*	Sector	Number of participants	Modality type of examination	Comparing body regions	Appropriateness definition	Rating	Intervention, (Control)	Methods Study design	Evidence level	Mono-/ multicenter	Data sources	Statistics
[62]	2006	UK	Adult	Inpatient	252	XR	–	National GL	Single	–	PC	3e	Mono	Clinical	Analytic
[63]	2010	USA	N/A	Outpatient	459	MRI, CT	Yes	National GL	N/A	–	RC	3e	Mono	Clinical	Analytic
[64]	2013	CAN	N/A	Inpatient	1901	MRI, CT	Yes	National GL	Single	–	RC	4a	Multi	Secondary	Descriptive
[23]	2018	SPA	Adult	Inpatient	2022	CT, XR	Yes	EU	Single	–	C–S	3e	Multi	Clinical	Analytic
[65]	2018	PAK	Adult	N/A	1205	CT	–	ACR	N/A	–	RC	3e	Mono	Clinical	Analytic
[66]	2019	AUS	Adult	Inpatient	109	XR	–	National GL	N/A	–	RC	3e	Mono	Clinical	Analytic
[67]	2020	ZAF	Adult	Inpatient	515	MRI, CT	–	ACR	N/A	–	RC	3e	Mono	Clinical	Analytic
[68]	2014	ZAF	Adult	N/A	219	MRI, CT	–	ACR	N/A	–	RC	4a	Mono	Clinical	Descriptive
[69]	2018	USA	Adult	Inpatient	88	MRI	Yes	ACR	Double independent	–	RC	4a	Mono	Clinical	Descriptive
[70]	2013	SPA	Adult	Outpatient, inpatient	584	MRI	–	EU	N/A	–	PC	3e	Multi	Primary	Analytic
[71]	2018	SPA	Adult	Outpatient	300	MRI	No	ACR	Single	–	RC	3e	Mono	Clinical	Analytic
[72]	2007	UK	N/A	Inpatient	121	CT	–	National GL	Consensus	–	RC	4a	Mono	Clinical	Descriptive
[22]	2020	POR	Adult	Inpatient	1427	CT, US	–	ACR	N/A	–	RC	3e	Mono	Clinical	analytic
[73]	2010	USA	Adult	Inpatient	2295	CT	–	ACR	Single	–	RC	4a	Mono	Clinical	Descriptive
[74]	2016	USA	Child	Inpatient	207	MRI, CT	–	ACR	N/A	–	RC	3e	Mono	Clinical	Analytic
[75]	2018	GHA	Adult	Outpatient	161	MRI	–	ACR	N/A	–	RC	4a	Mono	Clinical	Descriptive
[76]	2020	USA	Adult	Inpatient	1005	CT	–	National GL, ACR	Double independent	–	PC	3e	Mono	Clinical	Analytic
[77]	2012	USA	Adult	Inpatient	243	CT	–	National GL	Single	–	RC	3e	Mono	Clinical	Analytic
[78]	2011	ITA	Adult	Inpatient	500	CT, XR, US	–	National GL	N/A	–	RC	4a	Mono	Clinical	Descriptive
[79]	2013	USA	Child, adult	Inpatient	182	CT	–	Own	Single	–	RC	3e	Mono	Clinical	Analytic
[80]	2018	CAN	Adult	Inpatient	1087	MRI	Yes	National GL, ACR	Single	–	PC	4a	Multi	Clinical	Descriptive
[81]	2021	GHA	Child, adult, elderly	Outpatient, inpatient	11,806	CT	–	ACR	Double independent	–	RC	4a	Multi	Clinical	Descriptive
[82]	2016	USA	Adult	Inpatient	108	MRI, XR^1^	–	ACR	Single	–	RC	3e	Mono	Clinical	Analytic
[83]	2020	FIN	Child, adult	Inpatient	430	CT, XR	–	National GL	Single	Referral GL + education (B/A)	RC	2d	Mono	Clinical	Analytic
[84]	2002	UK	N/A	Inpatient	171	XR	–	National GL	Double independent	–	PC	4a	Mono	Clinical	Descriptive
[85]	2012	USA	Adult	Inpatient	1325	CT^2^, XR	–	ACR	Double independent	–	RC	4a	Mono	Clinical	Descriptive
[86]	2010	USA	N/A	Inpatient	251	CT	–	National GL, ACR	N/A	–	RC	4a	Mono	Clinical	Descriptive
[87]	2019	IRE	N/A	Inpatient	297	XR	–	National GL	Single	iRefer GL (B/A)	RC	2d	Mono	Clinical	Descriptive
[88]	2013	ITA	Adult	–	3950	MRI, XR, US	–	EU	N/A	–	RC	4a	Mono	Clinical	Descriptive
[89]	2017	ITA	N/A	Inpatient	13,941	CT, XR	–	ACR, EU	> 2 independent	–	RC	4a	Mono	Clinical	Descriptive
[90]	1994	CAN	N/A	Outpatient, inpatient	198	MRI	–	Own	N/A	–	RC	3e	Mono	Clinical	Analytic
[91]	2015	USA	ADULT	Inpatient	100	MRI, CT, XR	–	ACR	Single	–	RC	4a	Mono	Clinical	Descriptive
[92]	2020	CMR	Adult	Outpatient, inpatient	511	CT	–	ACR	Double independent	–	RC	3e	Mono	Primary + clinical	Analytic
[21]	2019	FRA, LUX, BEL	Child, adult (undefined)	Inpatient	718	MRI, CT	Yes	National GL	N/A	–	RC	4a	Multi	Clinical	Descriptive
[20]	2019	CHE	Adult	Inpatient	1997	XR	–	National GL, ACR	N/A	–	RC	3e	Multi	Clinical	Analytic
[93]	2006	USA	Adult	Inpatient	660	CT	Yes	ACR	N/A	–	RC	4a	Mono	Clinical	Descriptive
[94]	2016	GER	N/A	Outpatient, inpatient	265	Various (N/A)	–	National GL	N/A	–	RC	4a	Multi	Secondary	Descriptive
[95]	2003	GER	N/A	Outpatient	3079	Various (N/A)	–	Own	Double independent	–	PC	4a	Multi	Clinical	Descriptive
[96]	2021	AUS	N/A	Inpatient	642	CT, XR	–	ACR, EU	> 2 independent	–	RC	4a	Mono	Clinical	Descriptive
[97]	1997	USA	Adult	Inpatient	180	Various (N/A)	–	Own	Single	Radiological consultation (B/A)	PC	2d	Mono	Clinical	Descriptive
[98]	2013	USA	Adult	N/A	507	CT	–	National GL	Single	–	PC	4a	Mono	Primary	Descriptive
[99]	2014	USA	Adult, elderly	Inpatient	388	CT	–	ACR	N/A	–	RC	3e	Mono	Clinical	Analytic
[100]	2013	FIN	Adult, child	Inpatient	150	MRI	Yes	EU	Double independent	–	RC	4a	Mono	Clinical	Descriptive
[101]	2018	ITA	Adult	Inpatient	853	MRI, CT	Yes	ACR	Self-assessment	–	RC	3e	Multi	Clinical	Analytic
[103]	2014	UK	N/A	Inpatient	3085	MRI, CT	–	National GL	Double independent	–	RC	4a	Multi	Primary + clinical	Descriptive
[104]	2016	AUS	N/A	Inpatient	386	CT, XR, US	–	National GL	Double independent	–	RC	4a	Mono	Clinical	Descriptive
[105]	2013	CAN	N/A	Outpatient	1000	MRI	Yes	Own	N/A	–	PC	4a	Multi	Clinical	Descriptive
[106]	2020	USA	N/A	Inpatient	445	CT	–	National GL	N/A	Clinical decision making (B/A)	PC	2d	Mono	Clinical	Analytic
[111]	2017	CAN	Adult	N/A	2417	CT	–	National GL	N/A	–	RC	3e	Mono	Clinical	Analytic
[107]	2021	CAN	Adult	Inpatient	2577	CT	–	National GL	Single	AUC—implementation (B/A)	RC	2d	Mono	Clinical	Analytic

*ACR* American College of Radiology; *AUC* Appropriate use criteria; *AUS* Australia; *BEL* Belgium; *B/A* Before-after; *CAN* Canada; *CHE* Switzerland; *CMR* Cameroon; *CS* Cross-sectional; *CT* Computed tomography; *FIN* Finland; *FRA* France; *GER* Germany; *GHA* Ghana; *IRE* Ireland; *ITA* Italy; *LUX* Luxembourg; *MRI* Magnetic resonance imaging; *N/A* Not applicable; *PAK* Islamic Republic of Pakistan; *PC* Prospective cohort; *RC* Retrospective cohort; *SPA* Spain; *UK* United Kingdom; *US* Ultrasound; *USA* United States of America; *XR* Radiography; *y* Years; *ZAF* South Africa

^*^ (median; mean) in studies included: child = 0–18y, adult = 18–65y, elderly > 65y

^1^appropriateness results were provided for MRI solely

^2^appropriateness results were provided for radiography solely

Most of the studies referred to the definitions of appropriateness in guidelines (*question 1)*. Especially national guidelines (22/50) or the ACR AC (23/50) were used to define and judge appropriateness. Based on the applied guidelines, most of the studies provided dichotomous or ordinal ratings (e.g., appropriate, may be appropriate or not appropriate) to measure the appropriateness. Some studies did not distinguish between full, moderate, and no appropriateness and summarized the results densely into appropriate/not appropriate (*question 2)*.

Twenty-two out of 50 studies did not provide details about the methodology of the appropriateness ratings. If specified, a single reviewer (14/28) or at least two independent reviewers (12/28) undertook the ratings of appropriateness (*question 3).*

Five included studies (10%) were interventional studies [83, 87, 97, 106, 107]. The analyzed interventions included guideline implementations [83, 87, 107], radiological consultations [97] for residents and clinical decision support systems [106]. Two guideline implementations [83, 107] and clinical decision support [106] showed significantly improved appropriateness results. Radiological consultation did not reveal significant changes [97].

49 out of 50 studies were cohort studies with a majority of retrospective designs (39/50). Most were undertaken in monocentric (38/50) settings using clinical data (45/50) for most analyses *(question 4)*. For the most part, statistical results were reported descriptively (24/42). Applying Levels of Evidence (Levels of Evidence for Effectiveness) of the Joanna Briggs Institute, the majority of studies (*n* = 27/50) were rated with evidence level 4a (descriptive design) or 3e (*n* = 20/50) for uncontrolled studies [108].

A total of 42 studies provided detailed results of single modalities [20, 21, 23, 62–67, 69–77, 79–90, 92, 93, 98–107].

**Fig. 2:**
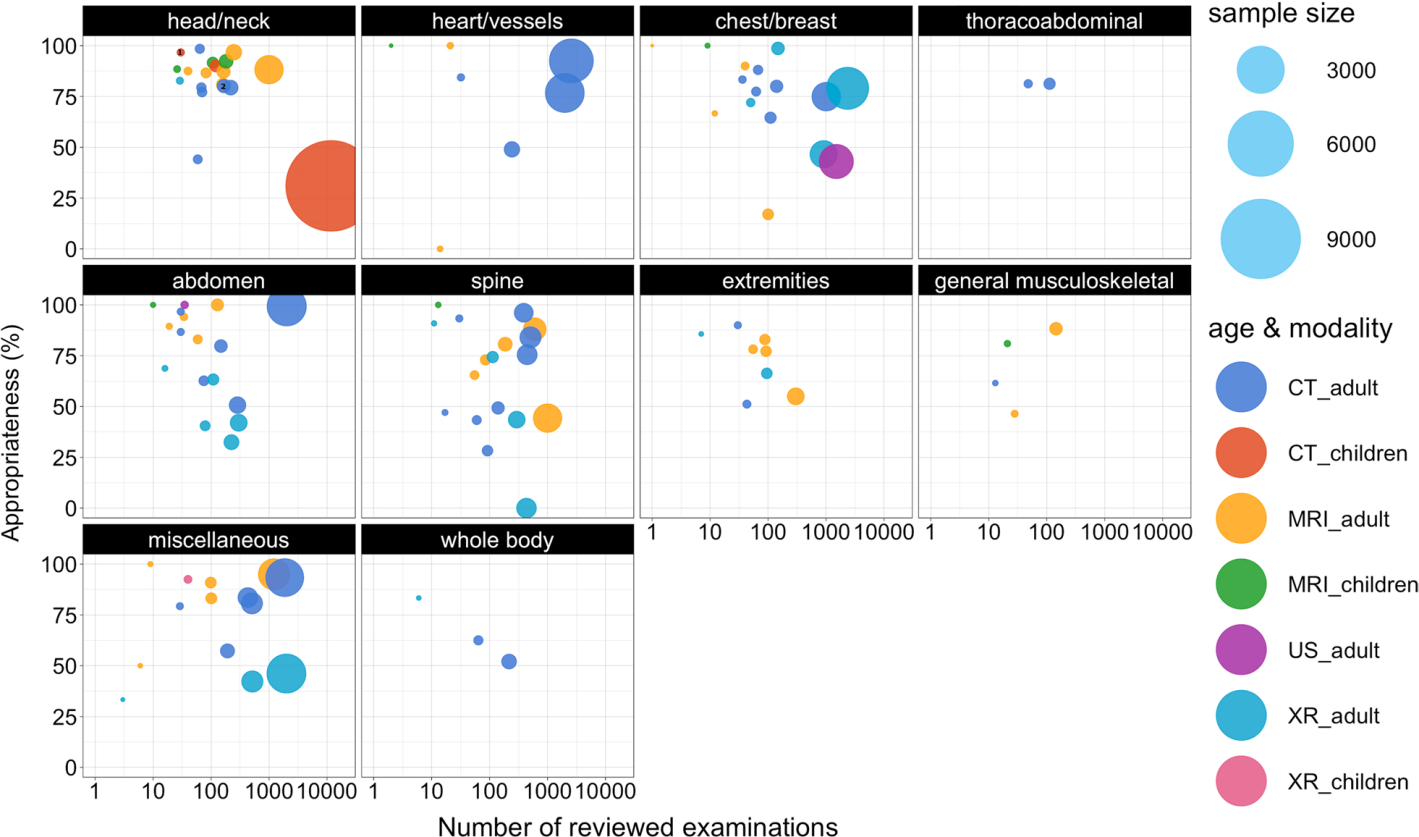
Bubble plot of study-individual appropriateness and number of reviewed examinations. The Bubble plot shows a matrix of study-individual appropriateness results (y-axis) and the number of reviewed examinations (x-axis). The age-stratified (adult, children) results were separately presented according to the aggregated body regions chest/abdomen/pelvis, chest/breast, general musculoskeletal (MSK)/extremities, head/neck, heart/vessels, other/miscellaneous, spine and whole body. Interventional studies are highlighted with black circles. Four studies reviewing the appropriateness of imaging other/miscellaneous body regions (CT + MRI) [64], heart/vessels CT [86], chest/breast CT [65] and whole body CT [89] were not included into the bubble plot due to missing information on the number of reviewed images. 1: Identical study results [100] of children and adult CT lead to a total overlap of bubbles in head/neck imaging. 2: Similar study results of adult MRI [93, 101] and adult CT [75] lead to a partial overlap of bubbles in head/neck imaging. *CT* Computed tomography. *MRI* Magnetic resonance imaging. *MSK* Musculoskeletal. *US* Ultrasound. *XR* Radiography

**Fig. 3:**
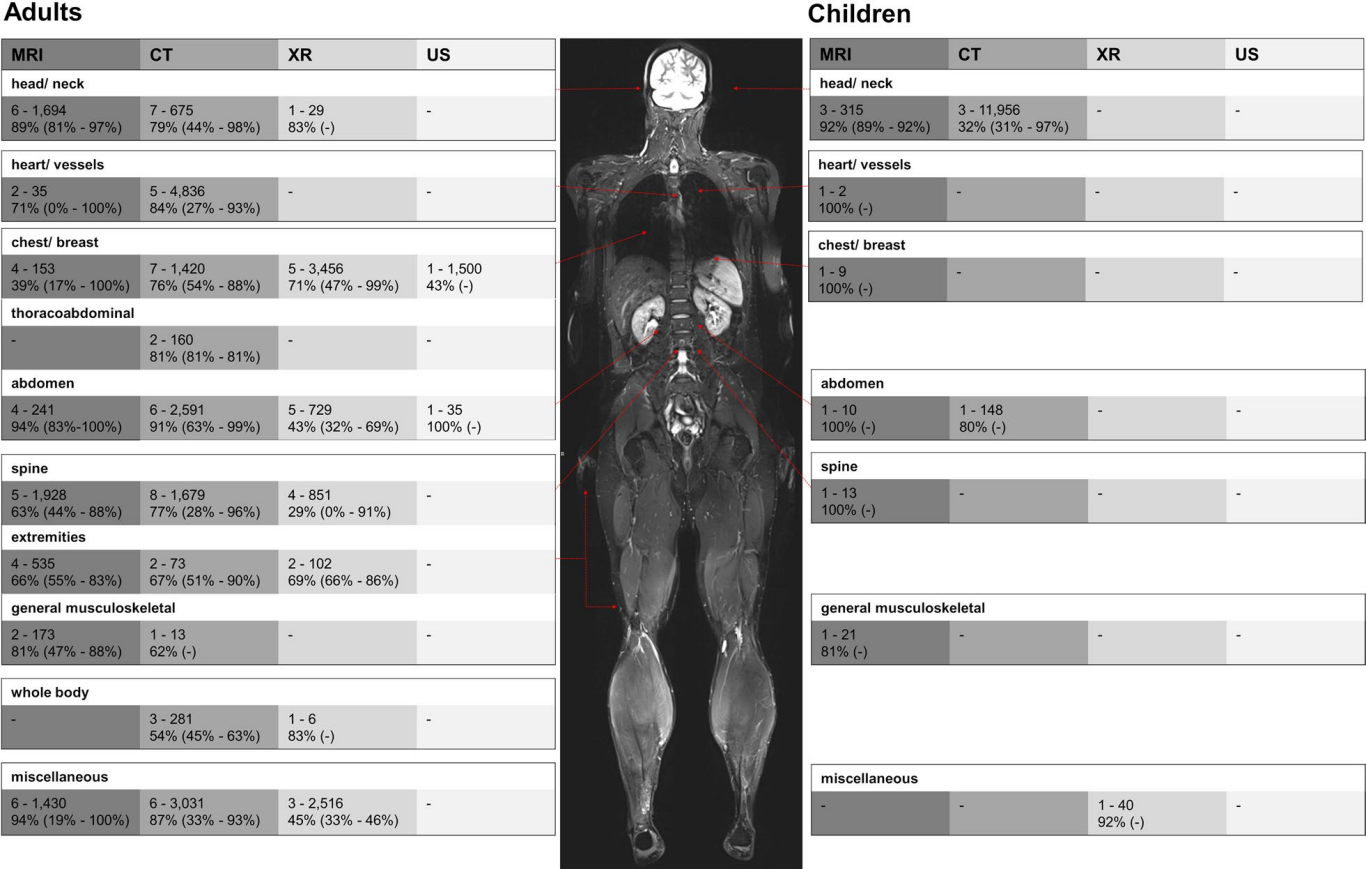
Appropriateness of MRI, CT, X-ray (XR) and ultrasound (US) in different body regions. The image shows the absolute number of studies and the accumulated number of reviewed examinations separated into different body regions. Based on the accumulated results, overall appropriateness (in %) was calculated presenting the span of study individual reported appropriateness in percent from lowest to highest. *Four studies reviewing the appropriateness of imaging other/miscellaneous body regions (CT + MRI) [64], heart/vessels CT [86], chest/breast CT [65] and whole body CT [89] reported the relative appropriateness without providing absolute numbers for different body regions. *CT* Computed tomography. *MRI* Magnet resonance imaging. *XR* Radiography. *US* Ultrasound

Seventeen studies encompassed 6559 MRI examinations [21, 63, 64, 67, 69–71, 74, 75, 79, 80, 82, 88, 90, 101, 103, 105] and reported an overall appropriateness of 79% (*n* = 5204/6559). 26 studies rated 26,715 CTs [21, 23, 63–65, 67, 72–74, 76, 77, 81, 83, 86, 89, 92, 93, 98–104, 106, 107] with an overall appropriateness of 60% (*n* = 16,363/27,309). Eleven studies [20, 23, 62, 66, 83–85, 87–89, 104] reported an appropriateness of 55% in 7729 reviewed radiographs (*n* = 4271/7729) and two studies [88, 104] reviewed 1535 radiological ultrasounds with an overall appropriateness of 44% (*n* = 680/1535). Overall, there are no patterns regarding size of study population and appropriateness (Fig. 2). The detailed study results were classified according to modalities and aggregated to body regions (Fig. 3), head/neck, chest, heart/vessels, abdomen, pelvis and musculoskeletal system, including spine and extremities, whole body and miscellaneous. Please refer to Additional file 1: Supplementary material 4 for the detailed study results (*question 2*).

### Head/neck

The appropriateness of head/neck imaging was analyzed by 14 studies [21, 63, 72, 74, 75, 79–81, 84, 93, 100, 101, 104, 105] reviewing MRI [21, 63, 74, 75, 79, 80, 101, 105], CT [21, 63, 72, 74, 81, 93, 100, 101, 104] and radiography [84]. They used national guidelines [21, 63, 72, 80, 84, 104, 105], ACR AC [74, 75, 80, 93, 101], EU guidelines [100] or internal/own guidelines [79].

Six studies focussing on MRI in adults [21, 63, 75, 80, 101, 105] reviewed 1694 examinations with 40 [63] to 1000 [105] records per study. 1500 (89%) head/neck MRIs were rated appropriate ranging between 81% (*n* = 130/161)[75] over 88% (*n* = 882/1000)[105] to 97% (*n* = 239/247) [80] study individual appropriateness. Three studies [74, 79, 80] reviewed 315 pediatric MRIs with an overall appropriateness of 92% ranging between 92 [74, 79] and 89% [80].

Nine studies reviewed 12,631 head/neck CTs [21, 63, 72, 74, 81, 93, 100, 101, 104] separable into 675 CTs of adults/unknown age in seven studies [21, 63, 72, 93, 100, 101, 104] and 11,956 pediatric CTs in three studies [74, 81, 100]. The appropriateness of head/neck CT in adults/patients of unknown age was rated with national guidelines [21, 63, 72, 104], EU guidelines [100] or ACR AC [93, 101]. It varied between 44% (*n* = 26/59) [63] and 98% (*n* = 63/64) [104], averaging out at an overall appropriateness of 79% (*n* = 532/675). Concerning pediatric head/neck imaging rated with on ACR AC [74, 81] or EU guidelines [100], the number of reviewed examinations and appropriateness varied between 97% (*n* = 29/30) [100], 90% (*n* = 108/120) [74] and 31% (*n* = 3660/11,806) [81]. Concerning head/neck radiographs, one study reported an appropriateness of 83% (*n* = 24/29) [84].

### Heart/vessels

Six studies about heart and/or vessel imaging [77, 80, 86, 101, 102, 107] were included. They reviewed 35 adult MRIs [80, 101], two pediatric MRIs [80] and 4836 adult CTs [77, 86, 101, 102, 107]. Two adult MRI studies report heterogeneous appropriateness rates of 100% (ACR AC: *n* = 21/21) [80] on the one and 0% (national GL: *n* = 0/14) on the other hand [101], leading to an overall appropriateness of 62% (23/37). One study reviewing two pediatric heart/vessel MRIs reported an appropriateness of 100% [80]. For CT, the overall appropriateness according to ACR AC [77, 101] or national guidelines [86, 102, 107] was 84% (*n* = 4052/4836). The number of reviewed CTs varied between 32 [101], 243 [77], 1984 [102] and 2577 [107]. The study without information on the number of reviewed examinations reported the lowest appropriateness of 27% (*n* = *N*/A) [86], and the study with the highest number of examinations reported the highest appropriateness: 93% (*n* = 2384/2577) [107].

### Chest/breast and thoracoabdominal imaging

13 studies analyzed chest/breast or thoracoabdominal imaging comprising 162 MRIs (adult: 153, children: 9) [21, 80, 88, 101], 1580 CTs [21, 23, 63, 65, 76, 93, 101], 3456 radiographs [23, 84, 88, 89, 104] and 1500 ultrasounds [88]. The overall appropriateness based on national guidelines [21, 23, 63, 65, 80, 84, 88, 104], EU guidelines [89] or ACR AC [76, 80, 89, 93, 101] varied between MRI (*n* = 69/162, 43%), CT (*n* = 1203/1580, 76%), radiography (*n* = 2462/3456, 71%) and ultrasound (*n* = 645/1500, 43%). Referring to individual study data, the numbers of reviewed examinations ranged from 1 [101] to 100 [88] MRIs, 36 [93] to 1005 [76] CTs and 50 [84] to 2350 [88] radiographs. The study-individual appropriateness results for MRI (17–100%), CT (54–88%) and radiographs (47–99%) varied either. Two studies did not provide information on the absolute number of reviewed images, with one study examining CTs (54% appropriate) [65] and the other examining radiography (no information on the number of images or results available) [89].

Thoracoabdominal imaging has been analyzed in two studies reviewing 160 CTs with an overall appropriateness of 81% [21, 63].

### Abdomen

Thirteen studies [21, 23, 62, 63, 66, 73, 80, 84, 87, 93, 100, 101, 104] analyzed the appropriate imaging of abdomen using ACR AC [73, 80, 93, 101], EU guidelines [100], or national guidelines [21, 23, 62, 63, 66, 80, 84, 87, 104].

MRI was analyzed in four studies [21, 63, 80, 101] reviewing 241 adult MRIs and 10 pediatric MRIs [80]. The overall appropriateness was found to be 94% for adults and 100% for children. The appropriateness varied between 83 [21] and 100% [80] in adult MRIs.

Six studies [21, 73, 93, 100, 101, 104] reviewed 2591 CT examinations (adult: 2443, children: 148) with an overall appropriateness rate of 89% (*n* = 2373/2591). These studies reviewed between 30 [100, 104] and 2022 [73] examinations. They reported appropriateness rates between 42 (*n* = 126/300) [87] and 99% (*n* = 2008/2022) [73] for adults and 80% (*n* = 118/148) for children in one study [101] reviewed with ACR [93, 101] or national guidelines [21, 73, 100, 104].

Radiography was analyzed by five (*n* = 729) [23, 62, 66, 84, 87] studies and ultrasound by one study (*n* = 35) [104], respectively. Overall, 311/729 (43%) radiographs were rated appropriate. Solely national guidelines were applied for the ratings. The number of reviewed radiographs per study (*n* = 16 [84]–*n* = 225 [62]) and results (32 [62]–69% [84]) varied. The 35 radiological ultrasounds were rated 100% appropriate according to national guidelines [104].

### Spine

Fourteen studies rated the appropriateness of spinal imaging [21, 63, 70, 80, 83–85, 87, 93, 98–100, 105, 106] including 1941 MRIs (adults: 1928, pediatric: 13) [21, 63, 70, 80, 105], 1679 CTs [21, 63, 83, 93, 98–100, 106], and 851 radiographs [83–85, 87]. Spinal MRI showed an overall appropriateness of 63% (*n* = 1221/1928) in six studies using ACR AC [80], EU guidelines [70] or national guidelines [21, 63, 80, 105]. However, the appropriateness in the studies ranged between 44 (*n* = 443/1000) [105] and 88% (*n* = 530/602) [70] in adults, and 100% (*n* = 13/13) in one study analysing pediatric MRIs [80]. Concerning spinal CTs, eight studies reported an overall appropriateness rate of 77% (*n* = 1292/1679) with varying results (28% [21]–96% [99]) and population (*n* = 17 [63]–*n* = 507 [98]) per study. Concerning radiography, both, population and appropriateness results varied between 0 (*n* = 0/433) [85], 74 (*n* = 84/113) [83] and 91 (*n* = 10/11) [84] leading to an overall appropriateness of 29% (*n* = 246/851).

### Extremities and general musculoskeletal imaging

Seven studies [21, 63, 69, 71, 84, 100, 104] reviewed extremity and general musculoskeletal imaging including 535 MRIs [21, 63, 69, 71], 73 CTs [21, 100], and 102 radiographs [84, 104]. The appropriateness ratings were based on ACR AC [69, 71], EU guidelines [100] or national guidelines [21, 63, 84, 104]. For MRI of extremities, the overall appropriateness rate was 66% (*n* = 352/535). The four underlying studies varied in sample size (55 [63]–300 [71]) and results (55 [71]–83% [69]). Two studies analysing CTs of extremities reported heterogeneous appropriateness rates of 51% (*n* = 22/43) [21] and 90% (*n* = 27/30) [100], resulting in an overall appropriateness of 67% (*n* = 49/73). The two studies including radiography of extremities, 68% of the radiographs (*n* = 69/102) were rated as appropriate with 86% (*n* = 6/7) [104] and 66% (*n* = 63/95) [84].

Two studies reviewed 194 MRIs (adults: 173, pediatric: 21) [80, 101] and 13 CTs [101] for general musculoskeletal imaging without further differentiation of body regions. Both referred to ACR AC and found appropriateness rates of 82% (*n* = 141/173) for adult MRIs [80, 101], 81% for pediatric MRIs (*n* = 17/21) [80] and 61% (*n* = 8/13) for CTs [101].

### Whole body imaging

Four studies [23, 89, 101] analyzed whole body imaging encompassing 281 CTs [23, 101] and 6 radiographs [84]. One study did not provide information about the number of reviewed CTs and reported an appropriateness rate of 45% [89]. The remaining two studies found an appropriateness rate of 52% (*n* = 113/217) [23] and 62% (*n* = 60/64) [101]. One study rated the appropriateness of six radiographs with 83% (*n* = 5/6) [84].

### Miscellaneous

Eleven studies did not classify body regions [20, 23, 63, 64, 67, 82–84, 90, 92, 103]. For adults, 1430 MRIs, [63, 64, 67, 82, 90, 103] 3031 CTs [23, 63, 64, 67, 92, 103] and 2516 radiographs [20, 23, 84] were reviewed. One study reviewed 40 miscellaneous pediatric radiographs [83]. According to ACR AC [67, 82, 92, 103], national [20, 23, 63, 64, 84], EU [83] or own guidelines [90], 94% of the MRIs (*n* = 1340/1430), 87% of CTs (2650/3031), and 45% (*n* = 1141/2516) of the radiographs were deemed appropriate. Among the studies, there was a broad variation in the number of reviewed examinations and resulting appropriateness rates with 6 (*n* = 3/6, 50%) [63] and 1215 (*n* = 1154/1215, 95%) [103] for MRIs, 192 (*n* = 110/192, 57%) [23] to 1870 (*n* = 1746/1870, 93%) [103] for CTs, and 3 (*n* = 1/3, 33%) [84] to 1977 (*n* = 922/1977, 46%) for radiographs [20]. One study that reviewed pediatric radiographs reported an appropriateness of 92% (*n* = 37/40) [83].

Eight studies [22, 68, 78, 91, 94–97] reviewed 6303 diagnostic examinations completely missing a specification of single body regions and three studies did not differentiate modalities [94, 95, 97]. Here, the overall appropriateness was 72% (*n* = 4548/6303). The number of reviewed examinations and resulting appropriateness rates varied between 52 (*n* = 50/52, 96%) [91] and 3079 (*n* = 2340/3079, 76%) [95].

One study about the appropriateness of CT examinations (33%) and MRI (*N*/A) did not provide absolute frequencies of the reviewed examinations and therefore did not enter the overall calculation and Fig. 2, as described above [64].

## Discussion

International radiological societies regularly define and update appropriateness criteria in order to improve quality, reduce unnecessary radiation exposure and reduce unnecessary costs [8]. An important step in achieving an efficient delivery of diagnostic imaging is to monitor the appropriateness rates. These rates are determined by the rate of by calculating the proportion of guideline-appropriate diagnostic imaging procedures to the total number of diagnostic imaging procedures performed. To date, no key performance indicators have been defined in this regard, and a robust methodology to derive them is indispensable.

This review presents several important new findings that are relevant to evaluate the appropriateness of radiological imaging in daily practice and research:

- More than 80% of the included literature relied on national or ACR AC to rate the appropriateness of diagnostic imaging. This emphasizes the importance of specific guidelines that can serve as a tool to rate appropriateness.
- All included studies reported appropriateness as a percentage. The benefit of a percentage is the applicability as key performance indicator. The studies presented a broad range of included examinations (88–11806) depending on the body region, the patient group and the modality.
- Many appropriateness ratings were methodically unclear (*n* = 22/50, 44%). Of the 28 studies reporting the rating methodology, less than 50% employed two independent reviewers. The data indicate that a small number of the included studies used double-independent appropriateness ratings. Double independent reading and justification is a common method in clinical medicine to reduce errors and identify discrepancies. It is also a standard methodological practice in systematic reviews and the coding of interviews in qualitative research [109–113]. Therefore, we assume that the raters who did use double-independent ratings had a higher level of awareness, as such a rating approach requires standardization and transparent a priori definitions [114]. Measurement of appropriateness was mainly based on guidelines but was aggregated completely or by multiple body regions in some studies [21, 103]. This simplification impedes a detailed analysis of concordance with indication-driven guidelines. Although guidelines seem to describe an imaging pathway clearly, one reader is not sufficient. One obstacle is that several studies found contradictory results as soon as 2 guidelines existed for one pathology [76, 84, 98, 99]. In order to compare the appropriateness on a national or even international scale, a harmonization among guidelines of different authorities is demanded. It was also shown that one guideline can lead to different decisions after an update [77, 83]. For this reason, a double-reading approach is needed to objectively evaluate the appropriateness of radiological imaging and the appropriateness must be rated in accordance with the timepoint of every single diagnostic imaging. A homogeneous aggregation of ACR ratings was used throughout this review to facilitate the comparison of different study results, which partially already were aggregated.
- According to the overall results of this review, 21% of the MRIs, 40% of the CTs, 44% of the radiographs, and 56% of the ultrasound examinations were not appropriate. Thus, thoroughly monitoring appropriateness rates bears high potential for resource management and radiation protection. At the same time, these appropriateness rates are key performance indicators for the gatekeeping function of radiologists.

For the sake of comparability, future studies or national/international audits should either apply a homogeneous aggregation or a separate specification of the appropriateness categories. Our review did not reveal patterns of study characteristics spawning high or low appropriateness rates, which further impedes a comparison between studies.

Assumingly, one reason for the great variety of appropriateness rates is the inhomogeneous application of guidelines in different indications and country/healthcare settings. Moreover, the analyses usually excluded non-codable and uncertain indications [115]. Including non-codable and uncertain indications would strengthen the reliability of appropriateness results. Thus, this knowledge should be exploited and not excluded from studies. The vast majority of studies reviewed inpatient settings, so a lack in the outpatient field must be stated. Importantly, investigation of appropriateness in pediatric populations was sparse, although children and adolescents are more vulnerable to x-ray exposure than adults. All these fields represent important future research topics.

After the creation of guidelines, the real implementation should be monitored. Blachar et al. found a substantial decrease in utilization of MRI and CT after the implementation of the ACR AC and the Royal College of Radiologist guidelines. They also reported a significant decrease in the costs paid by healthcare providers for CT and MRI [116]. More interventional studies could analyze how the implementation of guidelines affects appropriateness rates. Additionally, they would give more insights on the interdependence between the processes of diagnostic imaging, appropriateness, costs, medical treatment and outcomes.

Clinical decision support (CDS) tools are an emerging technique aimed at improving patient safety and promoting value-based imaging [117]. To our knowledge, the influence of a CDC on the appropriateness of radiologists has not been investigated as it was designed for referring providers [117]. According to a large randomized trial, CDS significantly reduced targeted imaging orders by 6%, but did not result in a significant change in the number of high- or low-cost scans. The authors of this study assume that CDS may lead to a modest improvement in the appropriateness of high-cost imaging [118].

Our review also has some limitations. Firstly, as with most scoping reviews, our analysis focused on the big picture with a high degree of aggregation and as a result, we may have missed specific details related to examinations that were rated as "maybe appropriate". Secondly, certain factors that could be associated with appropriateness, such as the quality of referrals, the nature of the disease (acute or chronic), and the type of disease or symptoms were not analyzed.

This review revealed heterogeneities in current evidence concerning study design, statistical methods, reporting and the appropriateness rating itself. This results in challenges regarding the appraisal of study validity due to small sample sizes, conflicting results and lack of reporting. Therefore, the following needs for further research and clinical practice can be derived from this review:

- An inevitable prerequisite for the measurement of appropriateness rates are evidence-based guidelines. Most studies used clinical data to assess the appropriateness of an imaging procedure. So far, clinical data entail difficulties as they are not standardised. As a consequence, clinical data sets are hard to compare within a single study and especially over several studies. Structured data might be a way forward to gain comparability and transparency. Furthermore, structured data or at least automated data are the very basis for intelligent tools like clinical decision support, which is requested by radiological societies such as the ESR [7, 11].
- A clear methodological pathway is needed for the measurement of appropriateness rates to obtain more valid data. In this review a high percentage of individual expert opinions were used to define appropriateness. International comparisons of appropriateness rates are impaired by the usage of different national guidelines.
- Thresholds should be defined while creating certain guidelines to establish awareness for both, the referrer and the radiologist. This could be applied to guidelines for very common indications. To discover applicable numbers, single and specific indications need to be subject of further studies. The design of these studies should include a large number of patients and elaborate specific key performance indicators for different modalities.

Based on this standardization, national audits as well as benchmarking of appropriateness in diagnostic imaging might be feasible. In summary, the following methodological standards should be met in future research or audits about imaging appropriateness to achieve a high level of evidence:

1. Implementation of multicentric studies, preferably with randomized controlled or interventional design;
2. Focus on clinical picture, not body regions, to create an inference to specific guidelines;
3. Consideration of the presence and quality of referrals for diagnostic imaging, as well as the existence of preliminary examinations;
4. Double independent readings of appropriateness;
5. High transparency regarding the rating results;
6. Analytical statistics with clearly defined influencing factors.

## Conclusion

In conclusion, many of the reported appropriateness rates might not be representative and cannot be taken as key performance indicators. More reliable and elaborate appropriateness rates with a valid methodical basis are needed. Especially the lack of knowledge about appropriateness rates in pediatric and outpatient imaging should be addressed. This review underlines the need of advanced research concerning appropriateness of clinical care in general and particularly of diagnostic imaging. Appropriateness and quality of indication in general reveal high importance for either medical care, clinical processes and quality.

## Supplementary Information

**Additional file 1.** **Supplementary material 1** - Original protocol registered at the Open Science Framework. **Supplementary material 2** - Applied search strategies. **Supplementary material 3** - Full text exclusions with reasons. **Supplementary material 4** - Appropriateness results per study.

## Data Availability

All data generated or analyzed during this study are included in this published article and its supplementary information files. Other data and materials not already published in the protocol, manuscript and supplement will be made available by the authors upon reasonable request.

## Abbreviations

ACR: American College of Radiology
ACR AC: American College of Radiology Appropriateness Criteria
CT: Computed tomography
ESR: European Society of Radiology
MRI: Magnetic resonance tomography
MSK: Musculoskeletal
OSF: Center for open science framework
PCC: Population–concept–context
US: Ultrasound
XR: Radiography
