# Supplementary material for: Measuring appropriateness of diagnostic imaging: a scoping review
Source: Insights Imaging. 2023 Apr 13;14:62. doi: 10.1186/s13244-023-01409-6 (PMC10102275; doi:10.1186/s13244-023-01409-6)
Supplement: Supplementary file 1 — Additional file 1. Supplementary material 1 - Original protocol registered at the Open Science Framework. Supplementary material 2 - Applied search strategies. Supplementary material 3 - Full text exclusions with reasons. Supplementary material 4 - Appropriateness results per study. [file 13244_2023_1409_MOESM1_ESM.pdf]

Electronic Supplementary Material

Measuring appropriateness of diagnostic imaging – a scoping review

Supplementary Material 1 – Original protocol registered at the Open Science Framework

1. Background and objective

What is it relevant to measure the quality of the determination of indication?

Medical services are resource limited, under budget and have a sensitive potential for complications. For medical services with particular risks of complications, it is essential that only those patients are treated who will benefit more than could be harmed. In order to ensure proper patient selection, it is necessary to communicate the patient's medical anamnesis (lat. indicare) between the different disciplines thoroughly to prevent under-, over or misuse. In radiology, overuse means that examinations are performed, for example, within short time intervals or without therapeutic implications and leading into an unnecessarily increased radiation exposure. In particular, overuse is critical for the risk of incidental findings, additionally to the radiation exposure. Accidental findings often lead to psychological distress during the investigation process and cause (unnecessary) follow-up costs.

How is indication quality measured?

Indication is basically a result of referral and clinical findings, which is a legal requirement for the justifying indication. Therefore, a simple indicator might be the existence of a referral. An existing referral should contain information that helps to choose the correct imaging modality, based on an evidence. Vice versa, more measurement parameters for indication quality can identify the number of not evidence based or even harmful diagnostic investigation (i.e. overuse). At the same time, the number of forborne diagnostic with evident benefit for the patient (i.e. underuse) could be compiled (ZEFQ, Volume 129, December 2017, Pages 37-40).

2. Research Questions

(i) What is the definition of appropriateness in radiological diagnostics in different study settings?
(ii) What are the measures of appropriateness in radiological diagnostics in different study settings?
(iii) Which methods are used to measure appropriateness in radiological diagnostics?
(iv) Which data are used to measure appropriateness in radiological diagnostics?

3. Methods

A Scoping Review will be conducted to answer the research questions using the updated guideline for Systematic Scoping Reviews developed by Joanna Briggs Institute [112]. *Scoping reviews* are mainly used when a research field is relatively unexplored and/or the literature is highly complex or heterogeneous which is why an orientation about the current state of the research literature (“map the available evidence”) should first be obtained (Khalil et al. 2016; Munn et al. 2018). Therefore, a critical appraisal of the studies included is generally not recommended and a synthesized answer of a precise question is not provided in Scoping reviews. The results are usually analysed by using descriptive statistical methods only and can then be visualized and presented by evidence mapping [112].

For the reporting of this Scoping review, the PRISMA-ScR Checklist will be used [18].

3.1 Inclusion and exclusion criteria based on the PCC framework

Table 1. Inclusion and exclusion criteria based on the PCC framework.

Criteria	Inclusion criteria	Exclusion criteria
**Population**	**Diagnostic**<br>-Patients undergoing radiological diagnostics<br>-patients of any age, (co)morbidity and sex	- Screening programs (e.g. breast, lung, prostate screening)<br>- radiotherapy (e.g. radiation)<br>- animal studies
**Concept**	- studies **analysing** appropriate and targeted use of radiological diagnostics<br>- studies **measuring** appropriate indication of radiological diagnostics	- clinical practice guidelines
**Context**	Single studies as well as aggregated evidence (systematic reviews, meta-analyses)	
**Publication type and language**	-Published journal articles available or articles not yet peer reviewed<br>- articles available as full text<br>- no language restrictions	Commentaries, case reports, conference papers,

3.2 Search strategy

A systematic literature search will be conducted in the databases *Medline*, *Embase* (via OVID), *Cochrane Central Register of Controlled Trials* and *Scopus*. In the development of a search strategy, pre-researched keywords, search terms and MESH terms were applied. On this basis, the following search string was designed for Medline and will be adapted to the database-specific features in the databases included. We also chose 5 Keypapers needed to be included into the database hits to validate our search strategy:

- Martins R, et al. Appropriateness of Radiology Test Requests by an Emergency Department: A Retrospective Study. Acta Med Port. 2020 Jan 3;33(1):7-14. doi: 10.20344/amp.12075. Epub 2020 Jan 3. PMID: 31928599.

- Cristofaro M, et al. Appropriateness: analysis of outpatient radiology requests. Radiol Med. 2012 Mar;117(2):322-32. doi: 10.1007/s11547-011-0725-2. Epub 2011 Sep 2. PMID: 21892709.

- Bouëtté A, et al. National audit on the appropriateness of CT and MRI examinations in Luxembourg. Insights Imaging. 2019 May 20;10(1):54. doi: 10.1186/s13244-019-0731-9. PMID: 31111303; PMCID: PMC6527721.

- Vilar-Palop J, et al. Appropriate use of medical imaging in two Spanish public hospitals: a cross-sectional analysis. BMJ Open 2018;8:e019535. doi: 10.1136/bmjopen-2017-019535

- Bertin CL, et al. Overuse of plain abdominal radiography in emergency departments: a retrospective cohort study. BMC Health Serv Res. 2019 Jan 14;19(1):36. doi: 10.1186/s12913-019-3870-2. PMID: 30642302; PMCID: PMC6332516.

<u>Search strategy Medline (Ovid)</u>

1	(Radiol*).ti,ab.
2	(diagnostic* or test* or request* or imag* or exam*).ti,ab. or Diagnostic Imaging/
3	(protocol* or pathwa* or practic* or standard* or guidel*).ti,ab or advance directive adherence/ or guideline adherence/ or "standard of care"/ or "health care quality, access, and evaluation"/
4	(Overus* or Appropriat* or inappropiat* or misus*).ti,ab. or Unnecessary Procedures/ or health services misuse/ or medical overuse/ or "meaningful use"/
5	(measu* or evaluat* or indicat* or predict* or analy*).ti,ab.
6	1 and 2 and 3 and 4 and 5

<u>Search strategy Embase (Ovid)</u>

1	(Radiol*).ti,ab.
2	(diagnostic* or test* or request* or imag* or exam*).ti,ab. or Diagnostic Imaging/
3	(protocol* or pathwa* or practic* or standard* or guidel*).ti,ab or practice guideline/ or clinical handover/ or clinical pathway/ or clinical protocol/ or good clinical practice/ or standard/
4	(Overus* OR Appropriat* OR inappropiat* or misus*).ti,ab.
5	(measu* or evaluat* or indicat* or predict* or analy*).ti,ab.
6	1 and 2 and 3 and 4 and 5

<u>Search strategy Scopus and CRCT</u>

1	Radiol*.ti,ab.
2	diagnostic* or test* or request* or imag* or exam*.ti,ab, keywords.
3	protocol* or pathwa* or practic* or standard* or guidel*.ti,ab, keywords
4	Overus* OR Appropriat* OR inappropiat* or misus*.ti,ab, keywords.
5	measu* or evaluat* or indicat* or predict* or analy*.ti,ab, keywords.
6	1 and 2 and 3 and 4 and 5

In addition, already published (systematic) reviews as well as the reference lists of the included articles (backward citation tracking) and articles in which these are cited (forward citation tracking via Scopus) will be screened.

3.3 Study selection process

After removing the duplicates using the literature management program Endnote, first the results will be checked by two independent reviewers at title and abstract level. Second, the remaining articles will be reviewed in full text. In case of diverging ratings of relevance on the full text level, a third person will be consulted with the aim of reaching a consensus. The open source software Rayyan (https://rayyan.qcri.org/) is used for this study selection process.

3.4 Extraction and synthesis of relevant content

Data extraction will be performed by one reviewer; its accuracy will be checked by another reviewer.

<u>General information (FS (i), (iv)):</u>
- Authors, year and nation of study conduct, language.
- Study design, study objective, measure of appropriateness
- Methodological categorization
- Categorization of used data sources (primary and/or secondary)
- Categorization of data (e.g. electronic health records, general surveillance data, primary data …)

- Number of participants or size of data sets
- Information on the quality of the used data (e.g. validation tests, representativeness, missing values)
- Evidence level

Specific information (FS (ii), (iii))
- (Suspected) diagnosis
- Care sectors (outpatient/inpatient, rehab)
- Age of the study participants
- Information on the comparison/control groups
- Outcome definition
- Outcome measures
- Discussion/ conclusion summary of results

Data extraction is done by 2 reviewers in a piloted standardized data extraction spreadsheet (Excel) using 5 reference articles to perform possible modifications before extracting all of the included studies.

3.5 Presentation of results

The research questions (i) and (ii) will be answered descriptively and, as far as possible, stratified according to countries, entity, health care sector and data source as well as study results. Based on these results, research gaps can be identified and described (cf. question (iii)).

4. Funding

No external funding

References

Khalil, H., Peters, M., Godfrey, C. M., McInerney, P., Soares, C. B., & Parker, D. (2016). An Evidence-Based Approach to Scoping Reviews. Worldviews Evid Based Nurs, 13(2), 118-123. doi:10.1111/wvn.12144

Munn, Z., Peters, M. D. J., Stern, C., Tufanaru, C., McArthur, A., & Aromataris, E. (2018). Systematic review or scoping review? Guidance for authors when choosing between a systematic or scoping review approach. BMC Medical Research Methodology, 18(1), 143. doi:10.1186/s12874-018-0611-x

Peter, M.D.J., Marnie, C., Tricco, A.C., Pollock, D., Munn, Z., Alexander, L., et al. (2020). Updated methodological guidance for the conduct of scoping reviews. JBI Evidence Synthesis, 18(10), 2119-2126. Doi:10.11124/jbies-20-00167

Tricco, A. C., Lillie, E., Zarin, W., O'Brien, K. K., Colquhoun, H., Levac, D., et al. (2018). PRISMA Extension for Scoping Reviews (PRISMA-ScR): Checklist and Explanation. Ann Intern Med, 169(7), 467-473. doi:10.7326/M18-0850

Supplementary Material 2 – Applied search strategies

#	Medline (Ovid)	Embase (Ovid)	Scopus	CRCT
**1**	Radiol*.ti,ab.	Radiol*.ti,ab.	Radiol*.ti,ab.	Radiol*.ti,ab.
**2**	(diagnostic* or test* or request* or imag* or exam*).ti,ab. or Diagnostic Imaging/	(diagnostic* or test* or request* or imag* or exam*).ti,ab. or Diagnostic Imaging/	diagnostic* or test* or request* or imag* or exam*.ti,ab, keywords.	diagnostic* or test* or request* or imag* or exam*.ti,ab, keywords.
**3**	(protocol* or pathwa* or practic* or standard* or guidel*).ti,ab or advance directive adherence/ or guideline	(protocol* or pathwa* or practic* or standard* or guidel*).ti,ab or practice guideline/ or clinical handover/ or clinical pathway/ or clinical protocol/ or good clinical practice/ or standard/	protocol* or pathwa* or practic* or standard* or guidel*.ti,ab, keywords	protocol* or pathwa* or practic* or standard* or guidel*.ti,ab, keywords
**4**	(Overus* or Appropriat* or inappropiat* or misus*).ti,ab. or Unnecessary Procedures/ or health services misuse/ or medical overuse/ or "meaningful use"/	(Overus* OR Appropriat* OR inappropiat* or misus*).ti,ab	Overus* OR Appropriat* OR inappropiat* or misus*.ti,ab, keywords.	Overus* OR Appropriat* OR inappropiat* or misus*.ti,ab, keywords.
**5**	(measu* or evaluat* or indicat* or predict* or analy*).ti,ab.	(measu* or evaluat* or indicat* or predict* or analy*).ti,ab.	measu* or evaluat* or indicat* or predict* or analy*.ti,ab, keywords.	measu* or evaluat* or indicat* or predict* or analy*.ti,ab, keywords.
**6**	1 and 2 and 3 and 4 and 5	1 and 2 and 3 and 4 and 5	1 and 2 and 3 and 4 and 5	1 and 2 and 3 and 4 and 5

Supplementary Material 3 – Full text exclusions with reasons

Reference	Reason
1. Beinvogl B, McSweeney M, Sabharwal S, Nurko S. Abdominal radiographs: Appropriately used in the management of functional constipation in children? Journal of Pediatric Gastroenterology and Nutrition 2016;63(Supplement 2):S90. doi: http://dx.doi.org/10.1097/01.mpg.0000503536.79797.66	Conference abstract
2. Salman L, Edwards Y, Majeed M. Adherence to diagnostic guidelines for suspected pulmonary embolism. American journal of respiratory and critical care medicine 2019;199(9).	Conference abstract
3. Sidhu M, Uthamalingam S, Lumish H, Engel LC, Hoffmann U, Abbara S, Brady TJ, Ghoshhajra BB. Adherence to the american college of cardiology foundation/society of cardiac computed tomography - 2010 appropriate use criteria guidelines by cardiologists in an academic tertiary health care institution. J Cardiovasc Comput Tomogr 2011;5(4 SUPPL. 1):S31-S32.	Conference abstract
4. Friedrich GJ, Maeser C, Hommel H, Pachinger O. The application of coronary computer tomography appropriateness criteria in clinical practice: A critical single center evaluation. European Heart Journal 2013;34(SUPPL. 1):845.	Conference abstract
5. Tang Y, Parai R, Pandya M, Patel L, Keller S. Are clinicians following the ACG or ACR guidelines when suspecting acute pancreatitis? American Journal of Gastroenterology 2012;107(SUPPL. 1):S110. doi: http://dx.doi.org/10.1038/ajg.2012.269	Conference abstract
6. Kelleher K, Ahmed I. An audit of abdominal x-rays; if their indications are appropriate, in particular for constipation, in a paediatric population. Irish Journal of Medical Science 2016;185(SUPPL. 5):S202-S203. doi: http://dx.doi.org/10.1007/s11845-016-1467-x	Conference abstract
7. M Chew CY. Clinical information supplied to radiologists, case note entries, and correlation with subsequent CT findings-A pilot study as part of a quality improvement project. J Med Imaging Radiat Oncol 2015;59(SUPPL. 1):79. doi: http://dx.doi.org/10.1111/1754-9485.12397	Conference abstract
8. Sarai P, Sebepos-Rogers G, Mann SD. CT pneumocolon in a district general hospital-is this test being used appropriately? Gut 2010;59(Supplement 1):A121-A122. doi: http://dx.doi.org/10.1136/gut.2009.209007j	Conference abstract
9. Gorey D, Umana E, Anderson T. Emergency department: Are we doing too many chest X-rays. Irish Journal of Medical Science 2017;186(6 Supplement 1):S252. doi: http://dx.doi.org/10.1007/s11845-017-1629-5	Conference abstract
10. Hextrum S, Ortiz J, Hornik J, Rosenblum J, Bar B. Emergent computed tomography angiography of intra-and extracranial vessels; Ordering patterns and yield at a single institution. Annals of Neurology 2017;82(Supplement 21):S100. doi: http://dx.doi.org/10.1002/ana.25024	Conference abstract
11. Enterline D, Rowley H. Evaluation of patients referred for neuroimaging: Which test and why? AM J ROENTGENOL 2011;196(5 SUPPL.):A226.	Conference abstract
12. Kalsy N, Webb J. Evaluation of the appropriateness of whole-body computer tomography (WBCT) in trauma patients in a tertiary trauma centre. Clinical Radiology 2014;69(SUPPL. 1):S23. doi: http://dx.doi.org/10.1016/j.crad.2014.05.097	Conference abstract
13. Fujimoto J, Karlin D, Ma J. Head imaging in the primary care setting: An analysis of ordering patterns, cost, and outcomes for minor head trauma, headaches, and migraines. Journal of Investigative Medicine 2015;63(1):188. doi: http://dx.doi.org/10.1097/JIM.0000000000000133	Conference abstract
14. Goiney C, Carlson B, Relyea-Chew A, Creutzfeldt C, Yuan C, Mossa-Basha M. Imaging appropriateness criteria may guide effective use of CT angiography in acute stroke workup. Stroke 2017;48(Supplement 1).	Conference abstract
15. Sheikh K, Belfi L, Baad M, Sanelli P. Imaging of acute blunt cervical spine trauma based on ACR Appropriateness Criteria. Emerg Radiol 2011;18(6):464. doi: http://dx.doi.org/10.1007/s10140-011-0991-2	Conference abstract
16. Yang T, Assaad M, Ananthasubramaniam K. Impact of implementing strategies to improve appropriate use of cardiac computed tomography angiography in clinical practice. J Cardiovasc Comput Tomogr 2013;7(SUPPL. 3):S67-S68.	Conference abstract
17. Carpeggiani C, Morales MA, Marraccini P, Mazzarisi A, Picano E. Inappropriateness of cardiovascular radiological imaging testing in a tertiary care referral center. European Heart Journal 2012;33(SUPPL. 1):247. doi: http://dx.doi.org/10.1093/eurheartj/ehs281	Conference abstract
18. Pollentine AA, Edey A, Chandratreya L. Introducing a cardiac CT service - Ensuring appropriate referrals and overcoming teething problems. Clinical Radiology 2012;67(SUPPL. 1):S18. doi: http://dx.doi.org/10.1016/j.crad.2012.06.093	Conference abstract
19. Ajayi OO, Hussain S. Is vetting CTPAs an appropriate use of radiologists' time? Clinical Radiology 2012;67(SUPPL. 1):S2. doi: http://dx.doi.org/10.1016/j.crad.2012.06.003	Conference abstract
20. Rupasinghe SN, Zikry MS, Marsden MR. Making emergency CT more efficient out of hours: Putting the surgeons in charge. International Journal of Surgery 2014;12(SUPPL. 3):S91.	Conference abstract
21. Bonner R, Barnewolt B, Halin N, Mostofi M. Portable chest x-ray utilization in the emergency department. Academic Emergency Medicine 2016;23(SUPPL. 1):S57. doi: http://dx.doi.org/10.1111/acem.12974	Conference abstract
22. Saccoia L, Jones DN, Quinn S, Ratcliffe J, Slavotinek J, Badiei A, Runciman W, Thomas M. Reducing the inappropriate use of medical imaging in the emergency department: A NHRMC TRIP Fellowship Project. J Med Imaging Radiat Oncol 2012;56(SUPPL. 1):4. doi: http://dx.doi.org/10.1111/j.1754-9485.2012.02422.x	Conference abstract
23. Figar S, Findakly S, Wong C, Du L. Retrospective audit of plain film imaging in acute ankle trauma: Are we choosing wisely? J Med Imaging Radiat Oncol 2017;61(Supplement 1):149-150. doi: http://dx.doi.org/10.1111/1754-9485.12657	Conference abstract
24. Cheng C, Brownlee R. A review of computed tomography imaging requests in the emergency department-Are automated approval systems the answer or part of the problem? J Med Imaging Radiat Oncol 2016;60(Supplement 1):14. doi: http://dx.doi.org/10.1111/17549485.12519	Conference abstract
25. Ngo D, Leong K, Marks G, Somasundaram A, Richards J, Du L. A single centre audit to assess clinical decision rules for imaging in suspected PE based on RANZCR guidelines. J Med Imaging Radiat Oncol 2016;60(Supplement 1):13. doi: http://dx.doi.org/10.1111/17549485.12519	Conference abstract

26. Atkinson L, Pratt S, Slevin T, Fritschi L, Catchpole B, Mendelson R, Fox R, Upton H, Deklerk N, Khong E. Towards appropriate use of diagnostic imaging in general practice. J Med Imaging Radiat Oncol 2009;53(SPEC. ISS. 1):A76. doi: http://dx.doi.org/10.1111/j.1440-1673.2009.01209.x	Conference abstract
1. Ranta A, Weatherall M, Gommans J, Tilyard M, Odea D, Dovey S. Appropriateness of general practitioner imaging requests for transient ischaemic attack patients: Secondary analysis of a cluster randomised controlled trial. J Prim Health Care 2017;9(2):131-135. doi: 10.1071/HC17005	Radiology/ referral request
2. Cristofaro M, Busi Rizzi E, Schininà V, Chiappetta D, Angeletti C, Bibbolino C. Appropriateness: Analysis of outpatient radiology requests. Radiol Med (Torino) 2012;117(2):322-332. doi: 10.1007/s11547-011-0725-2	Radiology/ referral request
3. Nawaz M, Amin A, Qureshi AN, Jehanzeb M. Audit of appropriateness and outcome of computed tomography brain scanning for headaches in paediatric age group. Journal of Ayub Medical College, Abbottabad : JAMC 2009;21(1):91-93.	Radiology/ referral request
4. van Schouwenburg F, Ackermann C, Pitcher R. An audit of elective outpatient magnetic resonance imaging in a tertiary South African public-sector hospital. S Afr J Radiol 2014;18(1). doi: 10.4102/sajr.v18i1.689	Radiology/ referral request
5. Krogh SB, Jensen TS, Rolving N, Laursen M, Thomsen JNL, Hansen CB, Werenberg CH, Rasmussen E, Carlson R, Jensen RK. Categorisation of lumbar spine MRI referrals in Denmark as compliant or non-compliant to international imaging guidelines: an inter-rater reliability study. Chiropr Man Thera 2021;29(1). doi: 10.1186/s12998-021-00370-9	Radiology/ referral request
6. Sodhi KS, Krishna S, Saxena AK, Sinha A, Khandelwal N, Lee EY. Clinical application of 'Justification' and 'Optimization' principle of ALARA in pediatric CT imaging: "How many children can be protected from unnecessary radiation?". EUR J RADIOL 2015;84(9):1752-1757. doi: 10.1016/j.ejrad.2015.05.030	Radiology/ referral request
7. Loughborough W. Development of a plain radiograph requesting algorithm for patients presenting with acute abdominal pain. Quant Imaging Med Surg 2012;2(4):239-244. doi: https://dx.doi.org/10.3978/j.issn.2223-4292.2012.09.06	Radiology/ referral request
8. Griffith JK, Borycki EM, Kushniruk AW. Diagnostic imaging ordering practices: physician perspectives and implications for decision support. Healthc Q 2014;17(2):62-70. doi: 10.12927/hcq.2014.23876	Radiology/ referral request
9. Poeran J, Mao LJ, Zubizarreta N, Mazumdar M, Darrow B, Genes N, Kannry J, Francaviglia P, Kennelly PD, Whitehorn J, Kilroy G, Garcia D, Mendelson DS. Effect of clinical decision support on appropriateness of advanced imaging use among physicians-in-training. AM J ROENTGENOL 2019;212(4):859-866. doi: 10.2214/AJR.18.19931	Radiology/ referral request
10. Costello JE, Shah LM, Peckham ME, Hutchins TA, Anzai Y. Imaging Appropriateness for Neck Pain. J Am Coll Radiol 2020;17(5):584-589. doi: 10.1016/j.jacr.2019.11.005	Radiology/ referral request
11. Bottari G, Stellacci G, Ferorelli D, Dell'Erba A, Arico M, Benevento M, Palladino G, Solarino B. Imaging Appropriateness in Pediatric Radiology during COVID-19 Pandemic: A Retrospective Comparison with No COVID-19 Period. Children (Basel, Switzerland) 2021;8(6). doi: https://dx.doi.org/10.3390/children8060463	Radiology/ referral request
12. Freeman R, Khanna S, Ricketts D. Inappropriate requests for magnetic resonance scans of the shoulder. Int Orthop 2013;37(11):2181-2184. doi: 10.1007/s00264-013-1968-4	Radiology/ referral request
13. Lee B, Mafi J, Patel MK, Sorensen A, Vangala S, Wei E, Sarkisian C. Quality improvement time-saving intervention to increase use of a clinical decision support tool to reduce low-value diagnostic imaging in a safety net health system. BMJ Open Qual 2021;10(1). doi: 10.1136/bmjoq-2020-001076	Radiology/ referral request
1. Reber J, McGauvran A, Froemming A. Abdominal radiograph usage trends in the setting of constipation: a 10-year experience. Abdom Radiol 2018;43(9):2231-2238. doi: 10.1007/s00261-018-1466-7	Appropriateness not measured
2. Martin TA, Quiroz FA, Rand SD, Kahn Jr CE. Applicability of American College of Radiology Appropriateness Criteria in a General Internal Medicine Clinic. AM J ROENTGENOL 1999;173(1):9-11. doi: 10.2214/ajr.173.1.10397090	Appropriateness not measured
3. Grassi R, Faggian A, Somma F, De Cecco CN, Laghi A, Caseiro-Alves F. Application of Imaging Guidelines in Patients With Foreign Body Ingestion or Inhalation: Literature Review. Semin Ultrasound CT MRI 2015;36(1):48-56. doi: 10.1053/j.sult.2014.10.004	Appropriateness not measured
4. Kelly AM, Kerr D. Are too many head CT scans ordered in emergency departments? Emerg Med 2000;12(1):50-54. doi: 10.1046/j.1442-2026.2000.00083.x	Appropriateness not measured
5. Manta A, O'Grady J, Bleakney R, Theodoropoulos J. Determining the appropriateness of requests for outpatient magnetic resonance imaging of the hip. Canadian journal of surgery Journal canadien de chirurgie 2019;62(4):224-226.	Appropriateness not measured
6. Gupta A, Ip IK, Raja AS, Andruchow JE, Sodickson A, Khorasani R. Effect of clinical decision support on documented guideline adherence for head CT in emergency department patients with mild traumatic brain injury. J Am Med Inform Assoc 2014;21(e2):e347-351. doi: 10.1136/amiajnl-2013-002536	Appropriateness not measured
7. Saadat S, Ghodsi SM, Firouznia K, Etminan M, Goudarzi K, Naieni KH. Overuse or underuse of MRI scanners in private radiology centers in Tehran. Int J Technol Assess Health Care 2008;24(3):277-281. doi: 10.1017/S0266462308080379	Appropriateness not measured

8. Blachar A, Tal S, Mandel A, Novikov I, Polliack G, Sosna J, Freedman Y, Copel L, Shemer J. Preauthorization of CT and MRI Examinations: Assessment of a Managed Care Preauthorization Program Based on the ACR Appropriateness Criteria® and the Royal College of Radiology Guidelines. J Am Coll Radiol 2006;3(11):851-859. doi: 10.1016/j.jacr.2006.04.005	Appropriateness not measured
1. Cullen EL, Aggarwal SR, Goss BC, Hodge DO, Gibbons RJ, Araoz PA. Comparison of the applicability of the 2006 and the 2010 cardiac CT angiography appropriate use criteria. J Am Coll Radiol 2013;10(4):258-267. doi: 10.1016/j.jacr.2012.07.014	Other study aim
2. Bairstow PJ, Mendelson R, Dhillon R, Valton F. Diagnostic imaging pathways: Development, dissemination, implementation, and evaluation. International Journal for Quality in Health Care 2006;18(1):51-57. doi: 10.1093/intqhc/mzi078	Other study aim
3. Cote DJ, Laws ER, Jr., Chicoine M, Hawlasi A, Dacey RG, Jr. The ethics of "Choosing wisely": The use of neuroimaging for uncomplicated headache. Clin Neurosurgery 2017;80(5):816-819. doi: 10.1093/neuros/nyw180	Other study aim
1. Taylor AJ, Cerqueira M, Hodgson JM, Mark D, Min J, O'Gara P, Rubin GD, Kramer CM, Berman D, Brown A, Chaudhry FA, Cury RC, Desai MY, Einstein AJ, Gomes AS, Harrington R, Hoffmann U, Khare R, Lesser J, McGann C, Rosenberg A, Schwartz R, Shelton M, Smetana GW, Smith SC, Wolk MJ, Allen JM, Bailey S, Douglas PS, Hendel RC, Patel MR, Shaw L, Stainback RF. ACCF/SCCT/ACR/AHA/ASE/ASNC/NASCI/SCAI/ SCMR 2010 appropriate use criteria for cardiac computed tomography: A report of the American college of cardiology foundation appropriate use criteria task force, the society of cardiovascular computed tomography, the American college of radiology, the American heart association, the American society of echocardiography. Circulation 2010;122(21):e525-e555. doi: 10.1161/CIR.0b013e3181fcae66	Guideline
2. Ford B, Dore M, Moullet P. Diagnostic Imaging: Appropriate and Safe Use. American family physician 2021;103(1):42-50.	Guideline
3. Carbonaro S, Villines TC, Hausleiter J, Devine PJ, Gerber TC, Taylor AJ. International, multidisciplinary update of the 2006 Appropriateness Criteria for cardiac computed tomography. J Cardiovasc Comput Tomogr 2009;3(4):224-232. doi: 10.1016/j.jcct.2009.05.010	Guideline
1. Miller JA, Williamson EE, Araoz PA, Raichlin E, McCully RB, Pellikka PA, Miller TD, Gibbons RJ, Hodge DO. Evaluation of Coronary CTA Appropriateness Criteria in an Academic Medical Center. J Am Coll Radiol 2010;7(2):125-131. doi: http://dx.doi.org/10.1016/j.jacr.2009.08.013	Duplet
2. Blachar A, Tal S, Mandel A, Novikov I, Polliack G, Sosna J, Freedman Y, Copel L, Shemer J. Preauthorization of CT and MRI Examinations: Assessment of a Managed Care Preauthorization Program Based on the ACR Appropriateness Criteria® and the Royal College of Radiology Guidelines. J Am Coll Radiol 2006;3(11):851-859. doi: 10.1016/j.jacr.2006.04.005	Duplet
1. Zucker EJ, Misono AS, Prabhakar AM. Abdominal Aortic Aneurysm Screening Practices: Impact of the 2014 U.S. Preventive Services Task Force Recommendations. J Am Coll Radiol 2017;14(7):868-874. doi: 10.1016/j.jacr.2017.02.020	Non-radiologic modality
1. Harpole LH, Khorasani R, Fiskio J, Kuperman GJ, Bates DW. Automated Evidence-based Critiquing of Orders for Abdominal Radiographs: Impact on Utilization and Appropriateness. J Am Med Informatics Assoc 1997;4(6):511-521. doi: 10.1136/jamia.1997.0040511	no results provided

Supplementary Material 4 – Appropriateness results per study.

Ref	**Overall**<br>*Guideline - modalities*	*categorization in case of no spec. body region*	**MRI**<br>*Age - GL - comparator (if given)*<br>*body region (study)*	*categorization*	**CT**<br>*Age - GL - comparator (if given)*<br>*body region (study)*	*categorization*	**Radiograph**<br>*Age - GL - comparator (if given)*<br>*body region (study)*	*categorization*	**Ultrasound**<br>*Age - GL - comparator (if given)*<br>*body region (study)*	*categorization*
[62]	-	-	-	-	-	-	Adult - RCR<br>abdomen: 32.4% (73/225)	abdomen/ pelvis	-	-
[63]	RBM<br>MRI/ CT: 74.0% (341/459)	-	N/A - RBM<br>spine: 65% (36/55)<br>shoulder: 63% (12/19)<br>brain/ orbits: 87% (35/40)<br>pelvis: 80% (4/5)<br>knee: 86% (31/36)<br>abdomen: 93% (13/14)<br>misc./ angiography: 50% (3/6)	Spine<br>extremities<br>head/ neck<br>abdomen/ pelvis<br>extremities<br>abdomen/ pelvis<br>other/ misc.	N/A - RBM<br>head/ brain: 38% (17/45)<br>maxillofacial 64% (9/14)<br>spine: 47% (8/17)<br>chest: 88% (59/67)<br>chest/ abdomen/ pelvis: 70% (7/10)<br>chest/ abdomen: 82% (84/102)<br>misc./ angiography: 79% (23/29)	head/ neck<br>head/ neck<br>spine<br>chest/ breast<br>chest/ abdomen/ pelvis<br>chest/ abdomen/ pelvis<br>other/ misc.	-		-	
[64]	CAR<br>MRI/ CT: 28% (91/325)*<br>*46% indeterminate	-	N/A - CAR<br>N/A: 19% (N/A)*<br>*54% indeterminate	other/ misc.	N/A - CAR<br>N/A: 33% (N/A)*<br>*41% indeterminate	other/ misc.	-	-	-	-
[23]	RP118<br>CT/ radiograph: 47.8% (967/2022)	-	-	-	Adult - RP118<br>body: 52.1% (113/217)<br>chest: 64.5% (71/110)<br>other: 57.3% (110/192)	whole body<br>chest/ breast<br>other/ misc.	Adult - RP118<br>chest/ thorax: 43% (315/733)<br>other: 42.2% (218/516)<br>mammography: 61.7% (108/175)<br>fluoroscopy: 40.5% (32/79)	chest/ breast<br>other/ misc.<br>chest/ breast<br>abdomen/ pelvis	-	-
[65]	-	-	-	-	Adult - ACR<br>chest: 55.4%	chest/ breast	-<br>-	-	-	-
[66]	-	-	-	-	-	-	Adult - DIP<br>abdomen: 63% (69/109)	abdomen/ pelvis	-	-
[67]	ACR<br>MRI/ CT: 81.4% (419/517)	-	Adult - ACR<br>N/A: 100% (9/9)	other/ misc.	Adult - ACR<br>N/A: 80.7% (410/508)	other/ misc.	-		-	
[68]	ACR - MRI/ CT: 93.6% (14/219)	other/ misc.	-		-		-	-		-
[69]	ACR - MRI: 83% (73/88)	-	Adult - ACR<br>foot, ankle: 93% (27/29)<br>knee: 77% (20/26)<br>leg, femur: 73% (11/15)<br>upper extremity: 83% (15/18)	extremities<br>extremities<br>extremities<br>extremities	-	-	-		-	
[70]	-	-	Adult - own*<br>lumbar spine: 88% (530/602)	spine	-	-	-	-	-	-
[71]	-	-	Adult - ACR<br>knee: 55% (165/300)	extremities	-	-	-	-	-	-
[72]	Various<br>CT: 77.0% (54/70)	-<br>-	-	-	N/A - NICE<br>head injuries: 100% (27/27)<br>seizures: 57% (4/7)<br>N/A - RCP<br>cerebrovascular accident: 76% (23/30)<br>N/A - BIS<br>meningitis: 0% (0/6)	head/ neck<br>head/ neck<br>head/ neck<br>head/ neck	-	-	-	-
[22]	ACR – US/CT/ MRI: 75.3% (1075/1427)	other/ misc.	-	-	-	-	-	-	-	-
[73]	-	-	-	-	Adult - ACR<br>enterography: 99.0% (2008/2022)	abdomen/ pelvis	-	-	-	-
[74]	ACR<br>MRI/ CT: 90.3% (187/207)*	-	Child - ACR<br>head/ neurotrauma: 91.6%, 95% CI, 86.3-96.9 (98/107)	head/ neck	Child - ACR<br>head/ neurotrauma: 90.0%, 95% CI, 84.6-95.4 (108/120)	head/ neck	-	-	-	-

Ref	**Overall**<br>*Guideline - modalities*	*categorization in case of no spec. body region*	**MRI**<br>*Age - GL - comparator (if given)*<br>*body region (study)*	*categorization*	**CT**<br>*Age - GL - comparator (if given)*<br>*body region (study)*	*categorization*	**Radiograph**<br>*Age - GL - comparator (if given)*<br>*body region (study)*	*categorization*	**Ultrasound**<br>*Age - GL - comparator (if given)*<br>*body region (study)*	*categorization*
	20 patients MRI + CT	-	-	-	-	-	-	-	-	-
[75]	-	-	Adult - ACR<br>brain: 81% (130/161)	head/ neck	-	-	-	-	-	-
[76]	-	-	-	-	Adult - ACCF vs. ACR<br>chest: 63.9% (640/1005) vs. 74.9% (753/1005)	chest/ breast	-	-	-	-
[77]	-	-	-	-	Adult - ACR 2006 vs. ACR 2010<br>coronary angiography: 14.8% (36/243) vs. 48.9% (119/243)	heart/ vessels	-	-	-	-
[78]	Nat. Law - CT/ US/ radiograph: 27.6% (138/ 500)	other/ misc.	-	-	-	-	-	-	-	-
[79]	-		Child – internal GL<br>head/ brain 92.3% (168/182)	head/ neck	-		-		-	
[80]	CAR-RG vs. ACR<br>MRI: 87.4% (730/835)<br>vs. 87.7% (685/787)	-	Adult - CAR-RG vs. ACR<br>brain: 96.8% (239/247) vs. 92.9% (221/238)<br>spine: 64.2% (129/201) vs. 80.6% (150/186)<br>muscoskeletal: 89.6% (129/144) vs. 88.3% (128/145)<br>abdomen/ pelvis: 100% (129/129) vs. 88.3% (99/119)<br>cardiac: 100% (21/21) vs. 71.4% (15/21)<br>breast: 90% (36/40) vs. 100% (34/34)<br>Child - CAR-RG vs. ACR<br>brain: 88.5% (23/26) vs. 83.3% (20/24)<br>spine: 100% (13/13) vs. 100% (9/9)<br>muscoskeletal: 80% (16/20) vs. 81% (17/21)<br>abdomen/ pelvis: 100% (10/10) vs. 100% (5/5)<br>cardiac: 100% (2/2) vs. 100% (1/1)<br>breast: 100% (9/9) vs. 100% (9/9)	head/ neck<br>Spine<br>general musculoskeletal<br>abdomen/ pelvis<br>heart/ vessels<br>chest/ breast<br>head/ neck<br>spine<br>general musculoskeletal<br>abdomen/ pelvis<br>heart/ vessels<br>chest/ breast	-	-	-	-	-	-
[81]	-	-	-	-	Child, adult, elderly - ACR<br>Head: 31% (3660/11806)	head/ neck	-	-	-	-
[82]	ACR - MRI: 83.3% (84/101)	other/ misc.	-	-	-	-	-	-	-	-
[83]	-	-	-	-	Adult - EU referral GL ‘07 vs. ‘09<br>lumar spine: 23% (7/30) vs. 63% (19/30), p=0.004<br>Child: N/A	spine	Adult - EU referral GL ‘05 vs. ‘09<br>lumar spine: 65% (39/60) vs. 85% (45/53), p=0.005<br>Child - EU referral GL ‘05 vs. ‘09<br>N/A 85% (17/20) vs. 100% (20/20)	spine<br>other/ misc.	-	-
[84]	-	-	-	-	-	-	N/A – RCR vs. clinical GL vs. RCR/ clinical GL<br>skull: 0% (0/24) vs. 8% (2/24) vs. 79% (19/24)<br>facial/ dental: 0% (0/5) vs. 0% (0/5) vs.100% (5/5)<br>cervical spine: 0% (0/6) vs. 17% (1/6) vs. 83% (5/6)<br>thoracic spine: 0% (0/3) vs. 0% (0/3) vs. 100% (3/3)<br>lumbar spine: 0% (0/2) vs. 0% (0/2) vs. 100% (2/2)<br>chest: 0% (0/50) vs. 28% (14/50) vs. 72% (36/50)<br>extremities: 0% (0/85) vs. 31% (26/85) vs. 65% (55/85)	head/ neck<br>head/ neck<br>spine<br>spine<br>spine<br>chest/ breast<br>extremities	-	-

Ref	**Overall**<br>*Guideline - modalities*	*categorization in case of no spec. body region*	**MRI**<br>*Age - GL - comparator (if given)*		**CT**<br>*Age - GL - comparator (if given)*		**Radiograph**<br>*Age - GL - comparator (if given)*		**Ultrasound**<br>*Age - GL - comparator (if given)*	
			body region (study)	*categorization*	*body region (study)*	*categorization*	*body region (study)*	*categorization*	*body region (study)*	*categorization*
							abdomen: 0% (0/7) vs. 0% (0/7) vs. 57% (4/7)<br>pelvis: 0% (0/9) vs. 22% (2/9) vs. 78% (7/9)<br>shoulder/ hip: 10% (1/10) vs. 10% (1/10) vs. 80% (8/10)<br>foreign body: 0% (0/6) vs. 17% (1/6) vs. 83% (5/6)<br>other: 0% (0/3) vs. 33% (1/3) vs. 33% (1/3)	abdomen/ pelvis<br>abdomen/ pelvis<br>extremities<br>whole body<br>other/ misc.		
[85]	-	-	-	-	-	-	Adult - ACR<br>cervical spine: 0% (0/433)	spine	-	-
[86]	-	-	-	-	N/A - ACCF<br>coronary angiography: 27% (46% indeterminate)	heart/ vessels	-	-	-	-
[87]	-	-	-	-	N/A - iRefer GL implementation - pre vs. post<br>spine: 48% (70/147) vs. 39% (58/147)<br>abdomen: 37.3% (56/150) vs. 46.6% (70/150)	spine<br>abdomen/ pelvis	-	-	-	-
[88]	-	-	Adult - national GLs<br>breast: 17% (17/100)	chest/ breast	-	-	Adult - national GLs<br>breast<br>79% (1857/2350)	chest/ breast<br>chest/ breast	Adult - national GLs<br>breast<br>43% (645/1500)	chest/ breast<br>chest/ breast
[89]	-	-	-	-	N/A - ACR, EU<br>whole body: 45% (N/A)	whole body	N/A - ACR, EU<br>chest: 38% (N/A)<br>Bedside chest: 45% (N/A)	chest/ breast<br>chest/ breast	-	-
[90]	-	-	N/A - own: Inside vs outside manitoba<br>MRI: 90% (90/99) vs. 76% (76/99)	other/ misc.	-	-	-	-	-	-
[91]	ACR - MRI/ CT/ radiograph: 96% (50/52)	other/ misc.	-	-	-	-	-	-	-	-
[92]	ACR – CT: 83.6% (361/432)	other/ misc.	-	-	-	-	-	-	-	-
[21]	GL of BEL, FR<br>CT: 61% (236/388)<br>MRI: 79% (261/330)		N/A - GL of BEL, FR<br>abdomen-pelvis: 83% (49/59)<br>extremities: 77% (71/92)<br>spine: 73% (62/85)<br>head-neck: 87% (71/82)<br>chest: 67% (8/12)	abdomen/ pelvis<br>extremities<br>spine<br>head/ neck<br>chest/ breast	N/A - GL of BEL, FR<br>abdomen-pelvis: 63% (47/75)<br>extremities: 51% (22/43)<br>spine: 28% (26/92)<br>chest-abdomen-pelvis: 81% (39/48)<br>head-neck: 79% (54/68)<br>chest: 77% (48/62)	abdomen/ pelvis<br>extremities<br>spine<br>chest/ abdomen/ pelvis<br>head/ neck<br>chest/ breast	-	-	-	-
[20]	-	-	-	-	-	-	Adult - various GL<br>HAS: 11.8% (235/1997)<br>ACR: 11.8% (235/1997)<br>DIP: 46.2% (922/1997)<br>iRefer: 46.2% (922/1997)	other/ misc.<br>other/ misc.<br>other/ misc.<br>other/ misc.	-	-
[93]	Trauma CT: 60.0%		-	-	N/A - ACR<br>head: 78% (108/139<br>spine: 49% (69/140<br>face: 91% (21/23<br>neck: 100% (4/4<br>chest: 83% (30/36<br>abdomen: 56% (80/143<br>pelvis: 45% (65/143	head/ neck<br>spine<br>head/ neck<br>head/ neck<br>chest/ breast<br>abdomen/ pelvis<br>abdomen/ pelvis	-	-	-	-
[94]	various (N/A): 71.3% (289/265)	other/ misc.	-	-	-	-	-	-	-	-
[95]	various (N/A): 76% (2340/3079)	other/ misc.	-	-	-	-	-	-	-	-
[96]	CT/ radiograph: 84% (538/642)	other/ misc.	-	-	-	-	-	-	-	-

Ref	**Overall**		**MRI**		**CT**		**Radiograph**		**Ultrasound**	
	Guideline - modalities	*categorization in case of no spec. body region*	*Age - GL - comparator (if given)*		*Age - GL - comparator (if given)*		*Age - GL - comparator (if given)*		*Age - GL - comparator (if given)*	
			body region (study)	*categorization*	*body region (study)*	*categorization*	*body region (study)*	*categorization*	*body region (study)*	*categorization*
[97]	Various (N/A) - IG vs. CG: 89.0% (13/119) vs. 55/61 (90%), n.s., p-value not given	other/ misc.	-	-	-	-	-	-	-	-
[98]	NEXUS/ CCR:<br>CT: 92.5% (469/507)		-	-	Adult - NEXUS vs. CCR<br>cervical spine: 84.0% (426/507) vs. 58.5% (297/507)	spine	-	-	-	-
[99]	-	-	-	-	Adult – NEXUS vs. CCR vs. NEXUS/CCR<br>cervical spine - 87.3% (339/388) vs. 64.1% (249/388) vs. 96.1% (373/388)	spine	-	-	-	-
[100]	Referral Crit. by EC<br>MRI: 93% (140/150)	-	-	-	Adult - Referral Crit. by EC<br>upper abdomen: 87% (13/15)<br>liver: 87% (13/15)<br>lumbar spine: 93% (28/30)<br>knee: 90% (27/30)<br>head: 97% (29/30)<br>Children – EC Referral Crit.<br>head: 97% (29/30)	abdomen/ pelvis<br>abdomen/ pelvis<br>spine<br>extremities<br>head/ neck<br><br>head/ neck - child	-	-	-	-
[101]	Adult - ACR<br>CT/ MRI: 81% (906/1122)	-	Adult - ACR<br>abdomen/ pelvis: 94.12% (32/34)<br>chest: 100.00% (1/1)<br>head: 87.20% (143/164)<br>musculoskeletal/ spine/ extremities: 46.43% (13/28)<br>vascular system: 0.00% (0/14)	abdomen/ pelvis<br>chest/ breast<br>head/ neck<br>general musculoskeletal<br>heart/ vessels	Adult - ACR<br>abdomen/ pelvis: 79.73% (118/148)<br>chest: 80.00% (112/140)<br>head: 79.36% (173/218)<br>whole body: 62.50% (40/64)<br>musculoskeletal/ spine/ extremities: 61.54% (8/13)<br>vascular system: 84.38% (27/32)	abdomen/ pelvis<br>chest/ breast<br>head/ neck<br>whole body<br>general musculoskeletal<br>heart/ vessels	-	-	-	-
[103]	ACR<br>CT/ MRI: 94% (2900/ 3085)		ACR<br>MRI: 95% (1154/1215)	other/ misc.	ACR<br>CT: 93% (1746/1870)	other/ misc.	-	-	-	-
[104]	ACEM/ DIP<br>CT/ radiograph/ ultrasound: 98.24% (279/284)	-	-	-	N/A - ACEM/ DIP<br>brain: 98.44% (63/64)<br>abdomen/ pelvis: 96.66% (29/30)	head/ neck<br>abdomen/ pelvis	N/A - ACEM/ DIP<br>chest: 99.16% (118/119)<br>chest mobile: 96.55% (28/29)<br>shoulder: 85.71% (6/7)	chest/ breast<br>chest/ breast<br>extremities	N/A - ACEM/ DIP<br>fetal: 100% (14/14)<br>pelvis: 100% (14/14)<br>scrotum: 100% (7/7)	abdomen/ pelvis<br>abdomen/ pelvis<br>abdomen/ pelvis
[105]	RAND - MRI:<br>66.2% (1325/2000)	-	N/A – RAND<br>lumbar spine: 44.3% (443/1000)<br>headache: 82.8% (882/1000)	spine<br>head/ neck	-	-	-	-	-	-
[106]	-	-	-	-	N/A - NEXUS – pre vs. post intervention<br>CT: 55% (23/42) vs. 78% (313/403), p=0.002	spine	-	-	-	-
[102]	-	-	-	-	Adult - AUC<br>cardiac CT: 76.7% (1522/1984)	heart/ vessels	-	-	-	-
[107]	-		-		Adult - AUC - pre vs. post intervention<br>Cardiac CT 87.4% (777/889) vs. 95.2% (1607/1688), p<0.001	heart/ vessels	-		-	

*notes:

ACCF - American College of Cardiology Foundation; ACEM - Australasian College for Emergency Medicine clinical guidelines; ACR - American College of Radiology; BIS - British Infection Society used for meningitis; CAR-RG - Canadian Association of Radiologists Referral guidelines; CAR-RG - Canadian Association of Radiologists Referral Guidelines; CCT - cranial CT; CT - computer tomography; CXR - chest x-ray; DIP - diagnostic imaging pathway; DIP - Diagnostic Imaging Pathways; EC - European Commission; HAS - Haute Autorité de Santé; MRI - magnetic resonance imaging; n.s. - not significant; N/A - not applicable; NEXUS - National Emergency X-Radiography Utilisation study; NICE - National Institute for Clinical Excellence used for head injuries and seizures; RBM - radiology benefits program; RCP - Royal College of Physicians used for Cerebrovascular accident; RCR - Royal College of Radiologists; RP118 - Radiation Protection 118; WB-CT - whole body computer tomography;
